# Pathomorphological characteristics of tuberculous placenta and its clinical implication

**DOI:** 10.1186/s13000-023-01419-4

**Published:** 2023-11-29

**Authors:** Zhidong Hu, Dong Zeng, Yuexiang Yang, Huijun Liu, Ao Wang, Duoduo Li, Min Liu, Yanling Feng

**Affiliations:** 1 Department of Scientific Research, Shanghai Public Health Clinical Center, Fudan University, Shanghai, China; 2 Department of Pathology, Shanghai Public Health Clinical Center, Fudan University, Shanghai, China; 3 Department of Pathology, The Fifth People’s Hospital of Puyang, Puyang, Henan Province China; 4 Department of Obstetrics and Gynecology, Shanghai Public Health Clinical Center, Fudan University, Shanghai, China

**Keywords:** Placenta, Tuberculosis, Pathological diagnosis, Acid-fast staining, Molecular pathology, Clinicopathology

## Abstract

**Background:**

The study of pathologic diagnosis of placental TB is rare. The aim of this study is analyzing the pathomorphological characteristics of tuberculosis (TB) placenta during pregnancy and its clinical significance.

**Methods:**

Nineteen cases of placental tissue specimens during pregnancy were collected from June 2015 to February 2022 at Shanghai Public Health Clinical Center, the only inpatient center for pregnant women with TB in Shanghai, China. Hematoxylin-eosin staining, acid-fast staining, and molecular testing were applied to analyze them comprehensively in combination with clinical information.

**Results:**

Among the 19 cases, 7 cases caused intrauterine stillbirth, 3 cases received artificial abortion required by the pregnant woman, the other 9 cases received standard delivery and the infants survived, however, 3 of them were low-weight preterm infants, and another 1 case suffered mild intrauterine asphyxia. The 9 surviving infants were followed-up, of which 3 cases got congenital TB. For pathological characteristics of placental tissues under light microscopy, there were 3 cases of epithelioid granuloma formation, 13 cases of acute fetal membranitis, 4 cases of caseous necrosis, 7 cases of inflammatory necrosis, 10 cases of coagulative necrosis, and 6 cases with small focal calcifications. All placental tissues were positive for acid-fast staining and polymerase chain reaction (PCR). Molecular pathological diagnosis showed that 18 cases were positive for Mycobacterium tuberculosis, with 1 case not having received examination.

**Conclusions:**

Combining acid-fast staining and molecular pathological testing is helpful for accurately diagnosing placental TB.

**Supplementary Information:**

The online version contains supplementary material available at 10.1186/s13000-023-01419-4.

## Background

Tuberculosis (TB), caused by *Mycobacterium tuberculosis* (*Mtb*), remains one of the major causes of infectious disease mortality. In 2020, it was estimated by the World Health Organization (WHO) that there were 10 million new TB cases and 1.4 million deaths globally [1], which is the first annual increase in the number of deaths since 2005. According to the latest Global Tuberculosis Report released by WHO, the trend of increased mortality continued in the year 2021 [2]. Pregnancy is a risk factor for active TB both in pregnancy and postpartum in women [3, 4]. The incidence of TB in pregnancy reflects the overall TB incidence, and there is a massive gap between developing and developed countries despite an annual decrease of 2%, the mass migration and tourism might lead to a resurgence of TB in pregnancy even in low-incident countries [5]. Thus, the incorrect diagnosis of TB during pregnancy and postpartum might increase the risk of perinatal death. Accurately diagnosing and treating active TB diseases during pregnancy and postpartum could reduce maternal and neonatal morbidities and mortality.

Currently, the routine diagnostics tools of TB include *Mtb* culture, acid-fast staining, GeneXpert MTB/RIF (Xpert), tuberculin skin test, interferon-gamma release assay, chest X-ray, pathological diagnosis, etc., all have their limitations [6, 7]. For example, as a golden standard, the traditional culture method needs about four weeks to yield a result, with a low sensitivity. Thus, the combinations of these available diagnostic tools are warranted to diagnose different statuses of TB diseases. However, the peculiarities of TB in pregnancy include the frequent absence of typical respiratory symptoms and the delay in diagnosis due to reluctance to undergo radiographic examination [8], as well as pregnancy itself may mimic and thus mask the early symptoms of *Mtb* infection such as fatigue and tachypnea [9], that aggravates the difficulties in diagnosing TB in pregnancy. Considering it was reported that placental infection is one of the effective manners for the acquisition of congenital TB by the neonate [10, 11], TB during pregnancy not only affects the physical and mental health of pregnant women but also affects the growth of the fetus [9, 12, 13]. Hence, TB pregnancy-specific diagnosis might provide additional valuable information for clinicians to analyze the intrauterine condition of the fetus.

Although the *Mtb*-infected placenta was regarded as an organ of great interest to pathologists at the beginning of the 20th century as an available source of TB pathogenesis study [14], the clinicopathology of placental tuberculosis has rarely been reported in recent years [11, 15, 16]. Herein, we collected nineteen cases with pathological diagnoses of placental TB. By combining hematoxylin-eosin (HE) staining, acid-fast staining, molecular testing, and clinical information, we provide a comprehensive analysis to improve the pathologists’ understanding of TB placenta and its pathologic profile, as well as provide a basis for clinicians to analyze the intrauterine condition of the fetus.

## Methods

### Study participants and specimens

This study was conducted at Shanghai Public Health Clinical Center, the only inpatient center for pregnant women with TB in Shanghai, China. In the past decade, most active TB patients in Shanghai during pregnancy or postpartum requiring hospitalization services were registered at this hospital. We screened the specimens of TB patients from June 2015 to February 2022 from the department of pathology, and a total of nineteen cases of placental tissue specimens during pregnancy were collected. In addition, HE staining, acid-fast staining, and polymerase chain reaction (PCR) were applied to comprehensively analyze these placental tissue samples in combination with clinical information. All patients were treated with standardized anti-TB treatment after diagnosis (Table 1). Written informed consent was obtained from the recruited participants or their immediate family members. This study was approved by the Ethics Committee of Shanghai Public Health Clinical Center.

**Table 1:** Clinical Characteristics of Study Participants

Case No.	Age	History of TB	Pregnancy information	CT diagnosis	Sputum result	Pathogenetic examination	Pregnancy outcome	Weeks of gestation*	Mode of delivery	Anti-TB treatment	Follow-up
1	28	N	Twin, IVF	Subacute hematogenous pulmonary TB	Smear+	AFB+, PCR+, T-spot+	Intrauterine stillbirth	27	Induced labour abortion	HREMfx	
2	30	Y	Single fetus, IVF	Obsolete pulmonary TB	Smear+	AFB+, PCR+	Intrauterine stillbirth	21	Spontaneous abortion	HRE	
3	31	N	Single fetus, spontaneous pregnancy	Acute miliary hematogenous disseminated pulmonary TB	Smear+, culture+	AFB+, PCR+, T-spot+	Intrauterine stillbirth	13	Induced labour abortion	HRZ	
4	39	Y	Single fetus, IVF	Acute miliary hematogenous disseminated pulmonary TB	Smear+	AFB+, PCR+, T-spot+	Surviving, mild intrauterine asphyxia	38	Cesarean	HRE	Infant with congenital TB
5	26	Y	Single fetus, spontaneous pregnancy	Pulmonary TB	Smear+	AFB+, PCR+	Surviving	38	Cesarean	HREZ	
6	32	N	Single fetus, IVF	Bilateral miliary hematogenous disseminated pulmonary TB	Smear+	AFB+, PCR+, T-spot+	Artificial abortion	21	Induced labour abortion	HRE	
7	33	N	Twin, IVF	Chronic pulmonary hematogenous disseminated TB	Smear+, culture+	AFB+, PCR+, T-spot+	Surviving, low-weight preterm infants	27	Natural labour	HRZ	One of the twins with congenital TB
8	30	N	Single fetus, spontaneous pregnancy	Bilateral pulmonary hematogenous disseminated TB	Smear+	AFB+, PCR+, T-spot+	Surviving, low-weight preterm infant	32	Cesarean	HREZ	
9	27	Y	Single fetus, IVF	Acute hematogenous disseminated pulmonary TB	Smear+	AFB+, PCR+	Intrauterine stillbirth	24	Spontaneous abortion	HRE	
10	40	N	Single fetus, IVF	Bilateral hematogenous disseminated pulmonary TB	Smear+	AFB+, PCR+	Artificial abortion	19	Induced labour abortion	HREZ	
11	27	N	Single fetus, spontaneous pregnancy	Bilateral hematogenous disseminated pulmonary TB	Smear+	AFB+, PCR+	Intrauterine stillbirth	26	Induced labour abortion	HRMfx + linezolid	
12	22	Y	Single fetus, spontaneous pregnancy	Bilateral miliary hematogenous disseminated pulmonary TB	Smear+	AFB+, PCR+, T-spot+	Surviving	31	Natural labour	HREZ	
13	36	U	Single fetus, IVF	N/A	Smear+	AFB+, PCR+, T-spot+	Surviving	36	Cesarean	HRZE	
14	35	U	Single fetus, spontaneous pregnancy	Bilateral acute hematogenous pulmonary TB	Smear+	AFB+, PCR+	Artificial abortion	16	Induced labour abortion	HREMfx + amikacin + meropenem	
15	28	Y	Single fetus, spontaneous pregnancy	N/A	Smear+	AFB+, PCR+	Surviving	39	Cesarean	U	
16	39	Y	Single fetus, spontaneous pregnancy	N/A	Smear+	AFB+, PCR+	Intrauterine stillbirth	19	Induced labour abortion	HRE	
17	31	Y	Single fetus, spontaneous pregnancy	Chronic inflammation	Smear+	AFB+, PCR+	Surviving	38	Cesarean	HRZE	
18	29	Y	Single fetus, IVF	Pulmonary TB	Smear+	AFB+, PCR+	Surviving, low-weight preterm infant	31	Cesarean	HREZ	Infant with congenital TB
19	36	N	Single fetus, spontaneous pregnancy	Acute hematogenous pulmonary TB	Smear+	AFB+, PCR+	Intrauterine stillbirth	20	Induced labour abortion	HREZ	

Abbreviations: Y: yes, N: no, U: unknown, IVF: in vitro fertilization, N/A: not available, PCR: polymerase chain reaction, H: isoniazid, R: rifampicin, E: ethambutol, Z: pyrazinamide, Mfx: moxifloxacin

* Weeks in which the fetus died or was delivered

The patients who met the following criteria were enrolled in the retrospective analysis study: [1] TB-positive cases during pregnancy. TB-positive were identified on the basis of sputum *Mtb* culture positivity or smear positivity, and was further confirmed by radiological or clinical syndromes, PCR amplification of the *Mtb* complex–specific gene *IS6110* (MeltPro, Zeesan Biotech) was used to exclude non-tuberculosis mycobacteria infection from culture/smear-positive cases; [2] The placenta tissue is *Mtb* PCR positive and AFB positive; and [3] the ability to provide detailed clinical history. Participants were excluded if they: [1] had incomplete clinical information; [2] HIV positive; and [3] Inherited genetic diseases found by genetic screening.

### Histological examination

All tissue specimens were fixed with 4% neutral formaldehyde, routinely dehydrated, paraffin-embedded, serially sectioned at 3 μm thickness, and stained with HE. Two pathologists independently performed the diagnosis following the Chinese expert consensus on the diagnosis of TB pathology in 2017.

### Acid-fast staining

Upon the hospital’s programmatic laboratory procedures, the Ziehl-Neelsen staining method for *Mtb* was carried out using kits (Baso Biotech, Zhuhai, China). Briefly, paraffin tissue sections were cut into 3 μm thick, dewaxed with conventional xylene, and dehydrated with gradient ethanol (high to low concentration). After washing with water, they were stained with the paraffin compound red for one hour. After washing, they were divided with hydrochloric acid ethanol for several seconds until no red color existed. Further, after washing, the samples were stained with methylene blue solution for 20 s, washed with water again, dehydrated with gradient ethanol and transparent with xylene, sealed with neutral gel, and observed with an oil microscope (×1000). Results interpretation: red rod-shaped, slightly curved, bead-like acid-resistant bacilli were defined as positive.

### Molecular pathological diagnosis using PCR method

The PCR diagnosis of TB was performed following the Chinese expert consensus on the diagnosis of TB pathology released in 2017. Briefly, the DNA extraction kit for paraffin-embedded tissue specimens was performed according to the manufacturer’s instructions (Tiangen Biochem, Beijing, China), and *Mtb* nucleic acid assay kit (DaAn Gene, Guangzhou, China) was used to detect *Mtb*-specific gene sequence *IS6110*. The *IS6110* gene is a 1191 bp repetitive insertion sequence that is usually present 6–20 times in the *Mtb* complex genome although fewer copy has been observed [17]. The oligonucleotide primers used were 5’-CCTGCGAGCGTAGGCGTCGG 3’ and 5’ CTCGTCCAGCGCCGCTTCGG 3’ [17]. The commercial *Mtb* nucleic acid assay kit (DaAn Gene, Guangzhou, China) showed a positive compliance rate higher than 99%, and negative compliance rate higher than 95%, compared with Sanger sequencing as the golden standard according to the kit’s instructions, and has authorized by the State Drug Administration of China to be used as TB DNA diagnosis kit, so no additional Sanger sequencing was performed in this study. The real-time fluorescence quantitative PCR instrument was a Roche Cobas Z480 automatic quantitative PCR analyzer. The kit was operated according to the kit’s instructions, with PCR amplification program is 98 °C for 3 min, followed by 45 cycles at 94 °C for 15 s, annealing of primers at 60 °C for 35 s, and machine cooling at 25 °C for 1 min. The Ct value less than 37 was considered positive.

## Results

### Characteristics of participants

A total of 19 patients were collected during the study period. These patients were aged 22–40, with a mean age of 30.4. Nine of 19 cases of placental TB had a history of TB diseases, the others denied TB infection history. CT examination indicated 15 cases of pulmonary TB lesions, including 12 cases of hematogenous disseminated pulmonary TB. Among the 19 cases, there were 7 cases of intrauterine stillbirth, 3 cases received artificial abortion which required by the pregnant woman, the other 9 cases received standard delivery and the infants were survived, however, 3 of the infants were low-weight preterm infants, and another 1 case suffered mild intrauterine asphyxia. Considering the genetic testing excluded the gene deficiencies that associated with inborn errors including Down’s syndrome and malformation, we assume the high rate of intrauterine stillbirth in our cohort might be caused by *Mtb* infection in the placenta. Interestingly, among the 19 cases of placental TB, 9 cases of pregnant women received in vitro fertilization, which were obviously higher than normal levels. In addition, among the 19 patients, there was 1 case of acute diffuse peritonitis with mechanical intestinal obstruction, 2 cases of TB meningitis, 1 case with viral hepatitis B, cholestasis of pregnancy, lupus erythematosus, larynx TB, and pelvic TB, respectively. All the patients had varying degrees of fever after delivery, with a temperature of 37.5–40.0 ℃. All patients were treated with standard anti-TB drug regimen. The details of the participants’ clinical characteristics and treatment regimen were shown in Table 1.

### Pathological histological examination and specific stain results

**Fig. 1:**
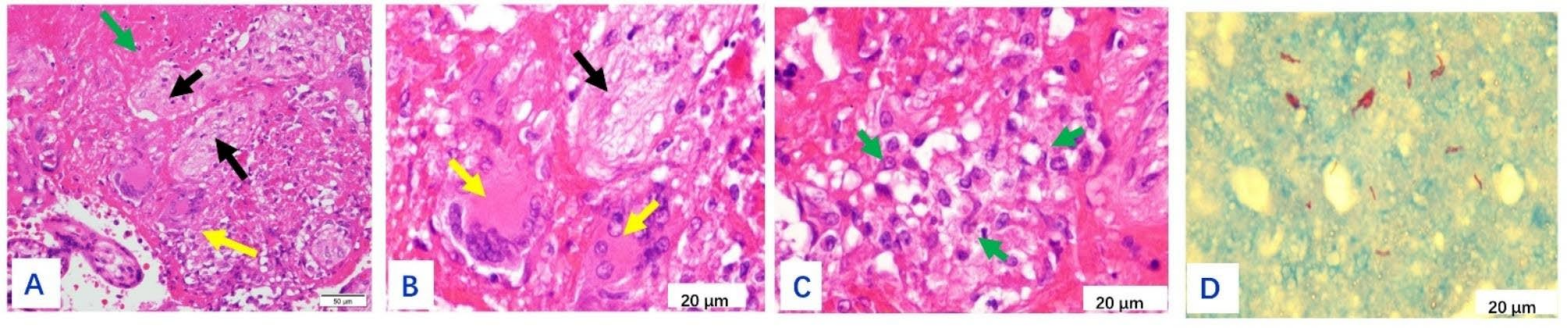
(**A**): Classic granuloma formation in placental tissue in case 8 (shown by yellow arrow), focal coagulative necrosis (shown by green arrow), partial fibrosis of villi (shown by black arrow). HE×400. (**B**): Enlargement of (**A**), yellow arrows showed granuloma multinucleated giant cells, and black arrows showed fibrotic villi. HE×1000. (**C**): Enlargement of (**A**), yellow arrow showed epithelioid cells in granuloma. HE×1000. (**D**): Acid-fast staining showed *Mycobacterium tuberculosis* with red color. ZN×1000

**Fig. 2:**
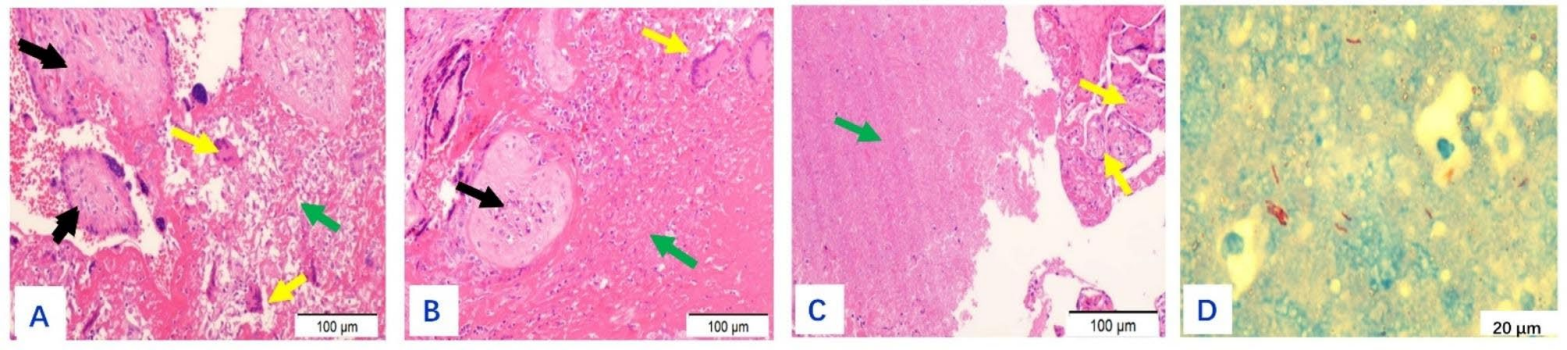
(**A**): Classic multinucleated giant cell formation in the placental tissue in case 10 (shown by yellow arrow), focal coagulative necrosis (shown by green arrow), part of the placental septum (shown by black arrow). HE×200. (**B**): Another view with a yellow arrow showing multinucleated giant cells, a black arrow showing placental septum, and a green arrow showing coagulative necrosis. HE×200. (**C**): Green arrow showed caseous necrosis, yellow arrow showed villi. HE×200. (**D**): Acid-fast staining showed the red color of *Mycobacterium tuberculosis*. ZN×1000

**Fig. 3:**
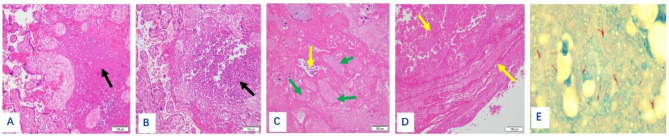
(**A**): Classic intra-placental focal inflammatory necrosis in case 19 (shown by black arrows). HE×200. (**B**): Purulent inflammation had shown by the black arrow. HE×200. (**C**): Focal piece of necrosis, necrotic villi (shown by green arrow), and small focal calcification (shown by yellow arrow). HE×200. (**D**): Caseous necrosis of fetal membranes and placenta necrosis (shown by yellow arrows). HE×200. (**E**): Acid-fast staining positive for *Mycobacterium tuberculosis* in red. ZN×1000

**Fig. 4:**
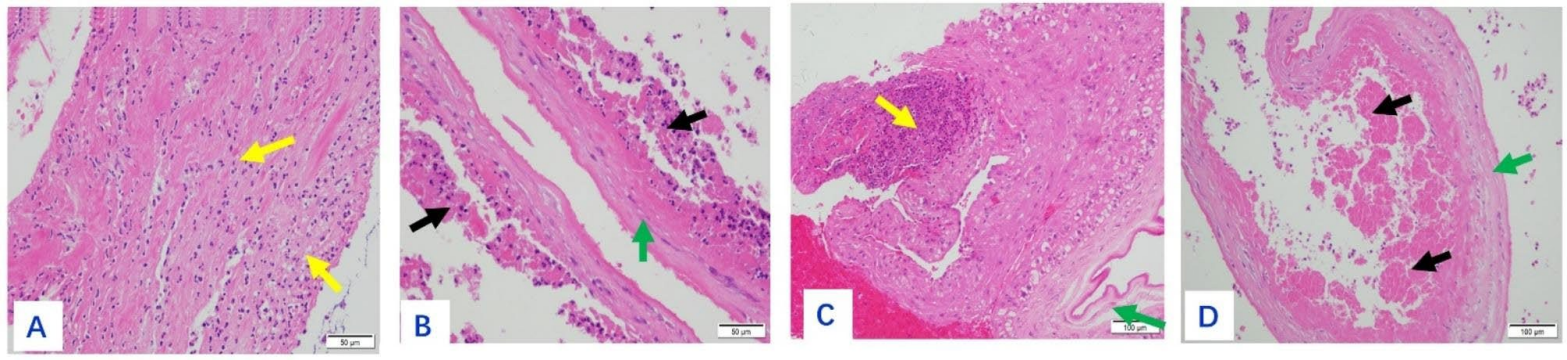
(**A**): Inflammatory cell infiltration in the fetal amnion, dominated by neutrophils (shown by yellow arrows). HE×400. (**B**): The amniotic membrane showed focal inflammatory necrosis (shown by black arrow), green arrow showed amniotic membrane that not necrotic yet. HE×400. (**C**): Purulent focus within the amniotic tissue (shown by yellow arrow), green arrow showed normal amnion. HE×200. (**D**): Partial caseous necrosis of the amnion (shown by black arrow), green arrow showed surviving amniotic tissue. HE×200

**Fig. 5:**
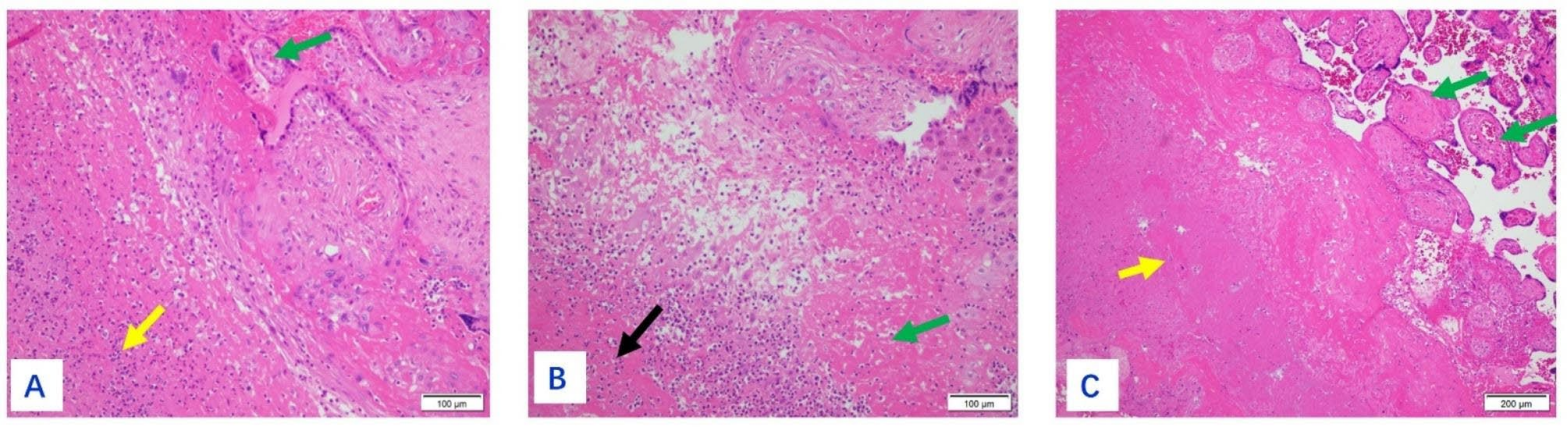
(**A**): A large number of neutrophils in the placental chorion (shown by yellow arrows), green arrow showed villi. HE×200. (**B**): Black arrow showed inflammatory necrosis, green arrow showed coagulative necrosis in the chorionic. HE×200. (**C**): Caseous necrosis in the chorionic membrane (shown by yellow arrow), green arrow showed villi. HE×100

The size of the placenta from the 19 puerperae ranged from 22 cm×14 cm×3.5 to 10 cm×7 cm×5 cm, with 1 umbilical cord attached to the fetal side and 3 umbilical vessels seen in the cut surface. The fetal side was bright blue and richly vascularized with the naked eye, while the maternal side was rough, lobulated, and dark red. Gray-white necrotic foci were seen in 7 cases, and calcified foci were seen in 2 cases. One case had an uneven thickness of the placenta, about 2.5 cm in the thick part and 0.5 cm in the thin part. A 6 × 6 × 2 cm of the fragmented placenta was seen in one case, with a soft gray-red texture. On light microscopy, the placenta was covered with trophoblast cells, the syncytiotrophoblast cells were aggregated, and fibrin deposits were seen around some of the villi. The representative staining results of normal mid- to late-stage placenta and normal fetal membrane tissue of health volunteers were shown in Supplementary Figures S1 and Figure S2, respectively. The representative staining results of placenta from tuberculous patients were shown in Figs. 1, 2, 3, 4 and 5, in which the classic granuloma formation, multinucleated giant cell formation, and intra-placental focal inflammatory necrosisin the placental tissue were shown, as well as the representative staining results indicating inflammatory cell infiltration in the fetal amnion and the outcome of the placental chorion were shown. Taken together, there were 3 cases of epithelioid granuloma formation, 13 cases of acute fetal membranitis, 4 cases of caseous necrosis, 7 cases of inflammatory necrosis, 10 cases of coagulative necrosis, and 6 cases with small focal calcifications (Table 2).

**Table 2:** The pathological characteristics of placental tissues under light microscopy

Case No.	Epithelioid granuloma formation	Acute fetal membranitis		Caseous necrosis	Inflammatory necrosis	Coagulative necrosis	Small focal calcifications
		Amnionitis	Chorioritis				
1	N	Y	N	N	N	Y	N
2	N	Y	N	N	N	Y	Y
3	N	Y	Y	N	Y	N	N
4	N	Y	N	Y	Y	N	N
5	N	Y	N	N	N	Y	Y
6	N	Y	Y	N	Y	N	N
7	N	Y	N	Y	N	Y	N
8	Y	Y	N	N	N	Y	Y
9	Y	Y	Y	N	Y	N	N
10	Y	Y	Y	Y	Y	N	N
11	N	N	N	N	N	Y	Y
12	N	N	N	N	N	Y	N
13	N	N	Y	N	N	N	N
14	N	N	N	N	N	Y	N
15	N	N	N	N	N	Y	Y
16	N	Y	N	N	N	Y	N
17	N	N	N	N	N	N	Y
18	N	N	N	N	Y	N	N
19	N	Y	Y	Y	Y	N	N
Total	3	13		4	7	10	6
		12	6				

Abbreviations: Y: yes, N: no

All placental tissues were positive for acid-fast staining, negative for PAS staining, and hexosamine silver. The pathological diagnosis of placental TB was combined with the medical history and its auxiliary examination.

### PCR results of placental tissue samples

All of 19 cases were positive for *Mtb* by PCR.

### Follow-up

As mentioned above, 9 cases received standard delivery and the infants survived, the 9 surviving infants were followed-up by telephone, of which 2 cases were lost follow-up, and 3 cases got congenital TB, which was relieved by anti-TB treatment.

## Discussion

TB in pregnancy is defined as the occurrence of TB in women during pregnancy, or the women of childbearing age (15–44 years) who develop pregnancy while untreated for TB, or the diagnosis of TB within three months after delivery [18, 19]. Women accounted for one-third of tuberculosis cases, with higher prevalence in the reproductive ages [20]. It was reported that active TB disease during pregnancy was associated with a significantly increased risk for poor maternal and fetal outcomes, including a 3-fold increase in maternal morbidity, 6-fold increase in perinatal death, 9-fold increase in miscarriage, 2-fold increase in preterm birth and low birth weight [21].

The pathogenesis of the high susceptibility of pregnant women to TB is mainly due to the increased levels of chorionic gonadotropin, estrogen and progesterone during pregnancy, which might disrupt the balance of the host immune system by inhibiting the proliferation and differentiation of immune cells, and then suppress the recognition and elimination of invading pathogens [22, 23]. In addition to hyperthyroidism, increased metabolic rate, and increased energy consumption; progesterone also can promote pulmonary capillary dilation, increased permeability, and pulmonary vascular congestion, which is conducive to the growth and reproduction of *Mtb* in the lungs [4, 24–26]. This leads to the easy spread of TB bacilli in the body from the lymphatic system to the circulatory system or directly into the blood system during newly *Mtb* infection, thus causing the spread of TB bacilli. In addition, considering the placenta was regarded as one of the major producers of endogenous progestogen, the spreading *Mtb* is easily resident into the placenta.

In this study, among the 19 cases of placental TB patients, there were 9 cases had a history of TB, the others denied TB infection history, indicating the TB infection history might be a risk factor for TB placenta. One of the possible reasons under this observation is that the host can not totally eradicate *Mtb* even through anti-TB chemotherapy due to the existence of TB granuloma [27, 28], thus, the long-lived *Mtb*, called “persisters”, can be existed even lifelong in cured TB patients. Thus, the secretion of high levels of endogenous progestogen during pregnancy might lead to the suppression of host immune systems and cause the reactivation of primary TB lesion or the resurgence of latent TB. Thus, the change in hormone levels during pregnancy might reactivate the long-lived “persisters” in the TB granuloma or caseous necrosis, causes the recurrence of latent TB lesions or initial foci of infection which is not entirely cleared by anti-TB drugs and the host anti-TB immune responses [29]. Herein, our data showed that there were 12 cases of acute miliary pulmonary tuberculosis. This indicates that most patients with placental TB are due to the spread of *Mtb* into the bloodstream to the placenta, and a few patients had pelvic or peritoneal spread. Unfortunately, none of these patients had biopsies of the fallopian tubes and endometrium to further assist in the corroboration.

The study of pathologic diagnosis of placental TB is rare. The typical features of TB lesion on light microscopy were granuloma formation with caseous necrosis. However, our study showed that there are only 3 cases of granuloma formation and 4 cases of caseous necrosis, indicating a delayed T cell-mediated hypersensitivity response in the host. In contrast, there were 13 cases of acute fetal membranes, all with predominantly neutrophil infiltration, suggesting that *Mtb* crossed the placental barrier, which was mediated by the innate immunity of the placenta in maternal blood. Of interest, the coexistence of acute fetal membranitis with different types of necrosis was shown in 12 cases, suggesting a delayed adaptive immune response in combination with an innate immune response, which were consistent with the findings of Carlos Abramowsky and Mana Taweevisit [11, 16]. Therefore, once acute chorioamnionitis, amnionitis, and focal necrosis of the placenta are detected, the patients should first be evaluated whether they were infected with TB. Differential diagnosis of pathology, especially granulomatous inflammation, mainly due to fungi, while excluding listeriosis, mycoplasma-infiltrated placentitis, combined with gram stain, antacid stain, PAS stain, hexosamine silver stain, immunohistochemistry, molecular pathological diagnosis, TB infection history, imaging, bacterial culture, T-SPOT, and other tests are necessary to make an accurate diagnosis.

In this study, there are as many as 9 cases of pregnant women received in vitro fertilization among the 19 cases of placental TB. One of the possible reasons is that genital TB in women of reproductive age is one of the risk factors causing infertility in high TB prevalence countries [30], thus, these populations tend to seek help through in vitro fertilization. Among the 9 patients received in vitro fertilization, the medical records showed that 4 had the history of TB, 2 had a history of prolonged low-grade fever but did not go for TB diagnosis and can not exclude *Mtb* infection, and the remaining 3 had endometriosis and polycystic ovary syndrome caused infertility. In addition, the use of high levels of progesterone during in vitro fertilization [31] is associated with immunosuppression as mentioned above [4, 24–26], which might lead to resurgence of TB diseases or even newly *Mtb* infection during pregnancy. In this cohort, the patients without TB diseases history might be considered as new infection, although we can not exclude the possibility or recurrent of latent TB infection if their primary TB lesion is naturally cured without chemotherapy. Thus, our study suggests that there is an association between in vitro fertilization and placental TB.

Regarding the effect of combined TB in pregnancy on the fetus, it has been suggested that effective anti-TB treatment is not only beneficial to the clinical cure of patients with mid-term pregnancy after 12 weeks of gestation but also has no significant adverse effects on the growth and development of infants. Failure to receive timely and effective treatment increases the risk of adverse maternal outcomes such as preterm delivery, miscarriage, neonatal congenital TB, and maternal or infant death [21]. In our study, there were 7 cases of intrauterine stillbirths. Placental TB was, at least partially, contributed to this outcome. Placental TB might cause some *Mtb* to enter the liver through the umbilical vein, causing the primary hepatic syndrome. A few *Mtb* enter the venous catheter through the umbilical vein and the lungs through the right atrium, causing the pulmonary primary complex. In 3 cases, the pregnancy was terminated due to fear of the effects of TB and drugs on the fetus, or the spread of TB bacilli from the placenta to the fetal membranes causing TB chorioamnionitis and/or amnionitis, aspiration of amniotic fluid by the fetus, and eventual death of the fetus due to severe infection. Nine fetuses survived, and after a medical history, these patients opted for anti-TB treatment with informed consent and continued the pregnancy, of which 3 were low birth weight preterm infants, including 1 case was mild intrauterine asphyxia. The 3 cases with positive T-SPOT-indicated neonatal TB (which was controlled by treatment) were followed-up. The rest of the cases were healthy for both mother and fetus, indicating severe TB diseases such as acute miliary pulmonary TB, acute TB peritonitis, combined cholestasis of pregnancy, hepatitis B virus-positive, etc. Thus, early and standardized anti-TB treatment benefits the mother and fetus.

## Conclusions

Placental TB in pregnancy is a rare pathological diagnosis, and granulomas and caseous necrosis are uncommon under light microscopy. In contrast, acute fetal membranitis and focal necrosis should not be ignored. Combining antacid staining and molecular pathology tests can help improve pathologists’ accurate diagnosis of placental TB. The pathological diagnosis of tuberculosis of placenta might provide a basis for clinicians to analyze the intrauterine condition of the fetus, and estimate the risk of intrauterine fetal death, intrauterine asphyxia, miscarriage, preterm birth, growth retardation, and neonatal TB.

### Electronic Supplementary Material

Below is the link to the electronic supplementary material.

Supplementary Material 1

## Data Availability

The datasets and images used during the current study are available from the corresponding author on reasonable request, and Shanghai Municipal Health Commission (202040330 to Dr. Min Liu).

## Abbreviations

TB: Tuberculosis
*Mtb*: *Mycobacterium tuberculosis*
WHO: World Health Organization
HE: hematoxylin-eosin
PCR: polymerase chain reaction
