# Supplementary material for: Pathomorphological characteristics of tuberculous placenta and its clinical implication
Source: Diagn Pathol. 2023 Nov 29;18:128. doi: 10.1186/s13000-023-01419-4 (PMC10685481; doi:10.1186/s13000-023-01419-4)
Supplement: Supplementary file 1 — Supplementary Material 1 [file 13000_2023_1419_MOESM1_ESM.docx]

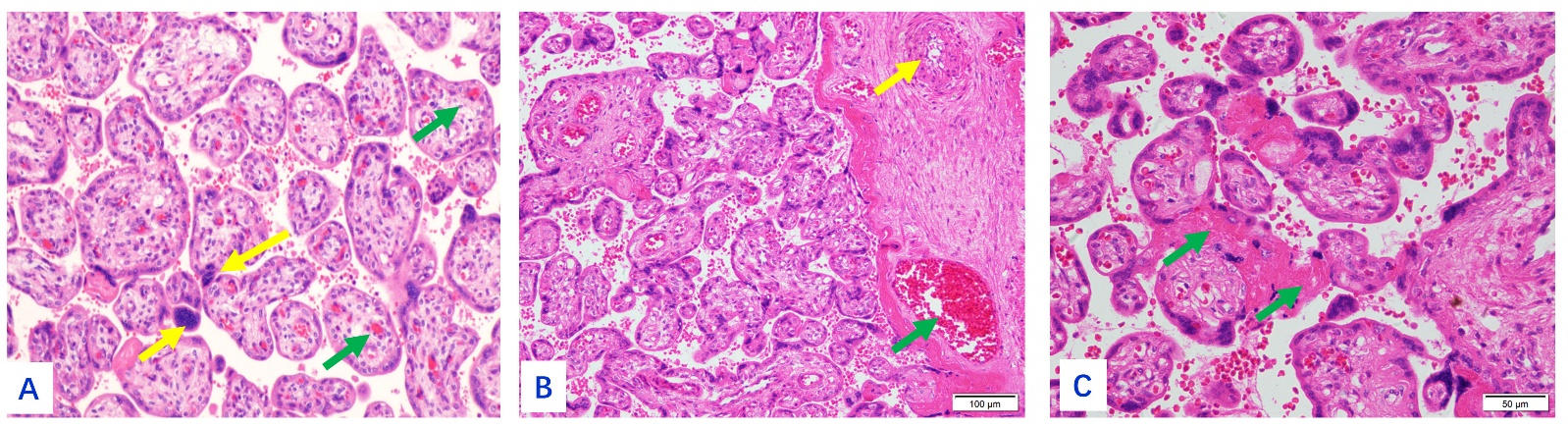


**Supplementary Figure S1.** (A): Normal mid- to late-stage placenta with abundant vascularity in the villi interstitial, shown by green arrows, and aggregation of [syncytial cells](javascript:;), shown by yellow arrows. Trophoblast cells can be seen. HE×200. (B): Umbilical vessels were branched in the normal mid- to late-stage placenta, with small veins shown by green arrows and small arteries shown by yellow arrows. HE×200. (C): Fibrin exudation was seen in the intervillous space in the normal mid- to late-stage placenta, shown by the green arrow. HE×400.


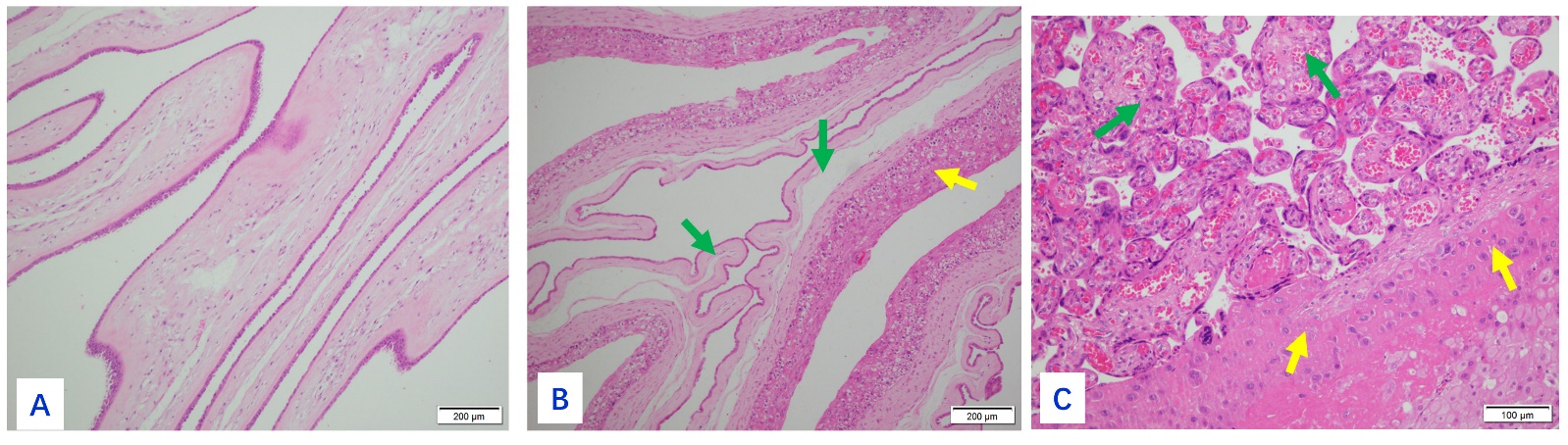


**Supplementary Figure S2.** (A): Normal fetal membrane tissue (amnion), composed of amniotic epithelium and fibrous connective tissue. HE×100. (B): Normal fetal membrane tissue, composed of amnion and smooth chorionic membrane, green arrow showed amnion, yellow arrow showed smooth chorionic membrane. HE×100. (C): Normal chorionic membrane covering the surface of the villi, fixing the villi and giving nutrients (yellow arrow showed chorionic membrane, green arrow showed villi). HE×200.
